# Integral Reinforcement-Learning-Based Optimal Containment Control for Partially Unknown Nonlinear Multiagent Systems

**DOI:** 10.3390/e25020221

**Published:** 2023-01-23

**Authors:** Qiuye Wu, Yongheng Wu, Yonghua Wang

**Affiliations:** School of Automation, Guangdong University of Technology, Guangzhou 510006, China

**Keywords:** adaptive dynamic programming, integral reinforcement learning, containment control, multiagent systems, neural networks

## Abstract

This paper focuses on the optimal containment control problem for the nonlinear multiagent systems with partially unknown dynamics via an integral reinforcement learning algorithm. By employing integral reinforcement learning, the requirement of the drift dynamics is relaxed. The integral reinforcement learning method is proved to be equivalent to the model-based policy iteration, which guarantees the convergence of the proposed control algorithm. For each follower, the Hamilton–Jacobi–Bellman equation is solved by a single critic neural network with a modified updating law which guarantees the weight error dynamic to be asymptotically stable. Through using input–output data, the approximate optimal containment control protocol of each follower is obtained by applying the critic neural network. The closed-loop containment error system is guaranteed to be stable under the proposed optimal containment control scheme. Simulation results demonstrate the effectiveness of the presented control scheme.

## 1. Introduction

Distributed coordination control of multiagent systems (MASs) has drawn expansive interest due to its potential application on agricultural irrigation [1], disaster rescue [2], microgrid scheduling [3], marine survey [4] and wireless communication [5]. The distributed coordination control aims to guarantee that all agents which exchange local information by communicating with their neighbors reach an agreement on some variables of interest [6]. Over the last decade, containment control has received increasing attention because of its remarkable performance in addressing the secure control issues, such as hazardous material treatment [7] and fire rescue [8]. The goal of containment control is to drive the followers to enter and keep within the convex hull spanned by multiple leaders. Numerous interesting and significant results of containment control have been presented. Reference [9] developed a fuzzy-observer-based backstepping control to achieve the containment of MASs. An adaptive funnel containment control was proposed in [10], where the containment errors converged to an adjustable funnel boundary. In practical applications, containment control has been developed for autonomous surface vehicles [4], unmanned aerial vehicles [11] and spacecrafts [12]. Notice that most of the aforementioned works have ignored the control performance with a minimum of energy consumption.

It is well-known that the Riccati equation or the Hamilton–Jacobi–Bellman equation (HJBE) are solved to acquire the optimal control for linear or nonlinear systems [13], respectively. In other words, the Riccati equation is a particular case of the HJBE. As a classical optimization algorithm, dynamic programming (DP) [14] is regarded as an effective way to obtain the optimal solution of the HJBE. However, as the dimension of state variables increases, the computation of the DP approach expands as a geometric series, which arouses the dilemma of the “curse of dimensionality”. With the success of AlphaGo, reinforcement learning (RL) has stimulated increasing enthusiasm from scholars to tackle the “curse of dimensionality” problem [15]. As is synonymous with RL, adaptive DP (ADP) [16] forward-in-time-solves the optimal control problem with the aid of neural network (NN)-based approximators. Moreover, ADP has been increasingly exploited for the optimal coordination control of MASs. Reference [17] established a cooperative policy iteration (PI) algorithm to solve the differential graphical games of linear MASs. In the nonlinear case, Reference [18] investigated the consensus problem via model-based PI with a generalized fuzzy hyperbolic critic structure. An event-triggered ADP-based optimal coordination control was proposed for the communication load and the commutation consumption was reduced [19]. To tackle the optimal containment control (OCC) problem, a finite-time fault-tolerant control was proposed via model-based PI [20]. In the presence of state constraints, Reference [21] presented a proper barrier function to transform the state constraint problem into an unconstrained case, thereafter the event-triggered OCC protocols were obtained. In Reference [22], distributed RL was applied to handle an OCC problem with collision avoidance of nonholonomic mobile robots. When the accurate model of the plant is not obtained, system identification is always employed. It should be pointed out that system identification is intractable for responding to dynamic changes of systems in time, which brings inevitable identification errors.

Recently, the integral RL (IRL) method was adopted to relax the accurate model requirement of the plant by constructing the integral Bellman equation [23,24]. An actor–critic architecture was adopted to execute the IRL algorithm, in which an actor NN learned the optimal control strategy and a critic NN was devoted to approximating the optimal value function. In the presence of heterogeneous linear MASs (HLMASs), the IRL method was developed to handle the robust OCC problem [25]. An adaptive output-feedback method was developed for the containment control for HLMASs via the IRL algorithm [26]. In Reference [27], the off-policy IRL-based OCC scheme was presented for unknown HLMASs with active leaders. However, the OCC problem of the nonlinear MASs with partially unknown dynamics has rarely been investigated via the IRL method. Moreover, the actor–critic architecture requires constructing the actor NN, which makes the control structure more complex. It is crucial to develop an IRL-based OCC scheme by implementing a simplified control structure. In addition, most of the aforementioned OCC approaches ensure the weight estimation error of the critic NN is uniformly ultimately bounded (UUB) only, which may degrade the control performance. All the above concerns motivated our research.

Inspired by the aforementioned works, we developed an IRL-based OCC scheme with asymptotically stable critic structure for partially unknown nonlinear MASs. The main contributions are reflected as follows.

(1) Different from existing control schemes [9,20], an IRL method is introduced to construct the integral Bellman equation without the system identification. Furthermore, IRL proves to be equivalent to model-based PI, which guarantees the convergence of the developed control algorithm.
(2) The IRL-based OCC scheme is implemented by a critic-only architecture for nonlinear MASs with unknown drift dynamics, rather than by an actor–critic architecture for linear MASs [25,26,27]. Thus, the proposed scheme simplifies the control structure.
(3) In contrast to the existing OCC schemes [20,21,22] which guarantee the weight errors to be UUB, a modified weight-updating law is presented to tune the critic NN weights, whose weight error dynamic is asymptotically stable.

This paper is organized as follows. In Section 2, graph theory and its application to the containment of MASs are outlined. In Section 3, the IRL-based OCC scheme and its convergence proof are presented for nonlinear MASs. Then, the stability of the closed-loop containment error systems is analyzed in detail. In Section 4, two simulation examples demonstrate the effectiveness of the proposed scheme. In Section 5, concluding remarks are drawn.

## 2. Preliminaries and Problem Description

### 2.1. Graph Theory

For a network with *N* agents, the information interactions among agents are reflected by a weighted graph G=(V,ε,A) with the nonempty finite set of nodes $V=\{\upsilon_{1},\ldots ,\upsilon_{N}\}$, the edge set ε⊆V×V and the nonnegative weighted adjacency matrix $A=[a_{ip}]$. If node $\upsilon_{i}$ links to node $\upsilon_{p}$, the edge $(\upsilon_{i},\upsilon_{p})\in \varepsilon$ is available with $a_{ip}>0$; otherwise, $a_{ip}=0$. For a node $\upsilon_{i}$, the node $\upsilon_{p}$ is named as a neighbor of $\upsilon_{i}$ when $(\upsilon_{i},\upsilon_{p})\in \varepsilon$. In this way, $N_{i}=\left\{\upsilon_{p}\in V:\left(\upsilon_{p},\upsilon_{i}\right)\in \varepsilon \right\}$ represents the set of all neighbors of $\upsilon_{i}$. Denote the Laplacian matrix as $L=D-A=[l_{ip}]$, where $D=\mathrm{diag}\{d_{11},d_{22},\ldots ,d_{NN}\}$, $d_{ii}=\sum_{p=1}^{N_{i}}a_{ip}$ and $l_{ip}$ satisfies

$$l_{ip}=\left\{\begin{array}{cc} \sum_{q=1,q\neq i}^{N_{i}}a_{iq},\;\;\; & i=p,\\ -a_{ip},\;\;\;\;\;\;\;\;\; & i\neq p. \end{array}\right.$$

It implies that each row sum of *L* equals to zero. A sequence of edges described by $\left(\upsilon_{1},\upsilon_{2}\right),\left(\upsilon_{3},\upsilon_{4}\right),\ldots$ with $\upsilon_{i}\in V$ is defined as a directed path. For arbitrary $(\upsilon_{i},\upsilon_{p})\in V$, a directed graph is strongly connected, if there is a directed path from $\upsilon_{i}$ to $\upsilon_{p}$, while the directed graph is said to contain a spanning tree if there exists a directed path from a root node to every other nodes with respect to G. This paper focuses on a strongly connected digraph with a spanning tree.

### 2.2. Problem Description

Consider the leader–follower nonlinear MASs in the form of the graph G with *M* leaders and *N* followers, where the node dynamic of the *i*th follower is modeled by

(1) $${\dot{x}}_{i}=f\left(x_{i}\left(t\right)\right)+g_{i}\left(x_{i}\left(t\right)\right)\mu_{i}\left(t\right),$$

where $x_{i}\in R^{n}$ is the state vector for the *i*th follower, $\mu_{i}\in R^{m}$ is the control input vector, i=1,2,…,N, and the nonlinear functions $f\left(x_{i}\right)\in R^{n}$ and $g_{i}\left(x_{i}\right)\in R^{n\times m}$ represent the unknown drift dynamic and the control input matrix, respectively. Denote the global state vector as $x={\left[x_{1}^{T},x_{2}^{T},\ldots ,x_{N}^{T}\right]}^{T}\in R^{N\times n}$.

**Assumption** **1.** *$f(x_{i})$ and $g_{i}\left(x_{i}\right)$ are Lipschitz continuous on the compact set $\Omega_{i}$ with f(0)=0 and the system *(1)* is controllable.*

Define the node dynamic of the *j*th leader as

(2) $${\dot{r}}_{j}=h_{j}\left(r_{j}\left(t\right)\right),$$

where $r_{j}\in R^{n}$ stands for the state vector of the *j*th leader, j=1,2,…,M and $h_{j}\left(r_{j}\right)\in R^{n}$ satisfies Lipschitz continuity.

**Definition** **1** (Convex hull [8]). *A set $C\subseteq R^{M\times n}$ is convex if for any $y_{1},y_{2}\in C$ and ∀ρ∈(0,1), $\left(\left(1-\rho \right)y_{1}+\rho y_{2}\right)\in C$. A convex hull of a finite set $Y=\{y_{1},y_{2},\ldots ,y_{M}\}$ is the minimal convex set, i.e., $Co\left(Y\right)=\left\{\sum_{j=1}^{M}\rho_{j}y_{j}\;|\;y_{j}\in Y,\rho_{j}\in R,\rho_{j}\geq 0,\sum_{j=1}^{M}\rho_{j}=1\right\}$.*

The containment control aims to find a set of distributed control protocols $\mu =\{\mu_{1},\mu_{2},\ldots ,\mu_{N}\}$ such that all followers stay in the convex hull formed by the leaders, i.e., $x_{i}\left(t\right)\to Co\left(Y\right)$ with $Y=\{r_{1},r_{2},\ldots ,r_{M}\}$. For the *i*th follower, the local neighborhood containment error $e_{i}$ is formulated as

(3) $$\begin{array}{cc} e_{i}=\; & \sum_{p\in N_{i}}a_{ip}\left(x_{i}-x_{p}\right)+\sum_{j=1}^{M}b_{ij}\left(x_{i}-r_{j}\right)\\ =\; & d_{ii}x_{i}-\sum_{p\in N_{i}}a_{ip}x_{p}+\sum_{j=1}^{M}b_{ij}\left(x_{i}-r_{j}\right), \end{array}$$

where $e_{i}\in R^{n}$, $b_{ij}\geq 0$ represents the pinning gain. Define $B_{j}=\mathrm{diag}\left[b_{1j},\ldots ,b_{ij},\ldots ,b_{Nj}\right]\in R^{N\times N}$. In fact, the connection between the *i*th follower and the *j*th leader is available if and only if $b_{ij}>0$. Denote the communication graph as $G_{x}=\left(G,x\right)$. The global containment error vector of $G_{x}$ is

$$e=\left(G⊗I_{n}\right)x+\left(\left(B\left(I_{M}⊗1_{N}\right)\right)⊗I_{n}\right)\bar{r},$$

where $e={\left[e_{1}^{T},e_{2}^{T},\ldots ,e_{N}^{T}\right]}^{T}\in R^{N\times n}$, $\bar{r}={\left[r_{1}^{T},r_{2}^{T},\ldots ,r_{M}^{T}\right]}^{T}\in R^{M\times n}$, $G=L+B(1_{M}⊗I_{N})$, $I_{n}$ represents the *n*-dimension identity matrix, $1_{M}$ stands for the *M*-dimensional column vector whose every element equals to 1 and $B=\left[B_{1},B_{2},\ldots ,B_{M}\right]\in R^{N\times NM}$. Considering (1), (2) and (3), for the *i*th follower, the local neighborhood containment error dynamic is formulated as

(4) $$\begin{array}{c} {\dot{e}}_{i}=\;F_{i}+c_{i}g_{i}\left(x_{i}\right)\mu_{i}+\sum_{p\in N_{i}}a_{ip}g_{p}\left(x_{p}\right)\mu_{p}, \end{array}$$

where $c_{i}=\left(d_{ii}+\sum_{j=1}^{M}b_{ij}\right)$ and $F_{i}=c_{i}f\left(x_{i}\right)-\sum_{p\in N_{i}}a_{ip}f\left(x_{p}\right)-\sum_{j=1}^{M}b_{ij}h_{j}\left(r_{j}\right)$. For the *i*th follower, the local neighborhood containment error is dominated not only by local states and local control inputs, but also by the information from its neighbors and the leaders. In order to implement the synchronization of the partially unknown nonlinear MASs (i.e., $e_{i}\to 0$), an IRL-based OCC scheme is designed in the next subsection.

## 3. IRL-Based OCC Scheme

### 3.1. Optimal Containment Control

For the local neighborhood containment error dynamic (4), define the cost function as

(5) $$J_{i}\left(e_{i}\left(0\right)\right)=\int_{0}^{\infty }P_{i}\left(e_{i}\left(\xi \right),\mu_{i}\left(\xi \right),\mu_{-i}\left(\xi \right)\right)d\xi ,$$

where $P_{i}\left(e_{i},\mu_{i},\mu_{-i}\right)=e_{i}^{T}Q_{i}e_{i}+\sum_{p\in \{N_{i},i\}}\mu_{p}^{T}R_{ip}\mu_{p}$ is a utility function, $\mu_{-i}=\left\{\mu_{p}|p\in N_{i}\right\}$ represents a set of the local control protocols from the neighbors of node $\upsilon_{i}$, and $Q_{i}\in R^{n\times n}$ and $R_{ip}\in R^{m\times m}$ are the positive definite matrices.

**Definition** **2** (Admissible control policies [17]). *The feedback control policies $\mu_{i}\left(e_{i}\right)\;\left(i\in I\right)$ are defined to be admissible with respect to *(5)* on a compact set $\Omega_{i}$, denoted by $\mu_{i}\left(e_{i}\right)\in A\left(\Omega_{i}\right)$, if $\mu_{i}\left(e_{i}\right)$ is continuous on $\Omega_{i}$ with $\mu_{i}\left(0\right)=0$, $\mu_{i}\left(e_{i}\right)$ stabilizes *(4)* on $\Omega_{i}$ and $J_{i}\left(e_{i}\left(0\right)\right)$ is finite $\forall e_{i}\left(0\right)\in \Omega_{i}$.*

**Definition** **3** (Nash equilibrium [17]). An N-tuple admissible control policy $\mu^{*}\left(e\right)=\{\mu_{1}^{*}\left(e_{1}\right),$$\mu_{2}^{*}\left(e_{2}\right),\ldots ,\mu_{N}^{*}\left(e_{N}\right)\}$ is said to constitute a Nash equilibrium solution in graph $G_{x}$, if the following N inequalities are satisfied

$$\begin{array}{c} J_{i}\left(e_{i},\mu_{i}^{*},\mu_{-i}^{*}\right)\leq J_{i}\left(e_{i},\mu_{i},\mu_{-i}^{*}\right),\;\;\;i=1,2,\ldots ,N, \end{array}$$

where $\mu_{-i}^{*}=\left\{\mu_{1}^{*},\ldots ,\mu_{i-1}^{*},\mu_{i+1}^{*},\ldots ,\mu_{N}^{*}\right\}$.

This paper aims to find an *N*-tuple optimal admissible control policy $\mu^{*}\left(e\right)$ to minimize the cost function (5) for each follower such that the Nash equilibrium solution in $G_{x}$ (i.e., the OCC protocols) is obtained.

For arbitrary $\mu_{i}\left(e_{i}\right)\in A\left(\Omega_{i}\right)$ of the *i*th follower, define the value function

(6) $$C_{i}\left(e_{i}\left(t\right)\right)=\int_{t}^{\infty }P_{i}\left(e_{i}\left(\xi \right),\mu_{i}\left(\xi \right),\mu_{-i}\left(\xi \right)\right)d\xi .$$

When (6) is finite, then the Bellman equation is

(7) $$\begin{array}{c} 0=\;e_{i}^{T}Q_{i}e_{i}+\sum_{p\in \{N_{i},i\}}\mu_{p}^{T}R_{ip}\mu_{p}+\nabla C_{i}^{T}\left(e_{i}\right)\left(F_{i}+c_{i}g_{i}\left(x_{i}\right)\mu_{i}+\sum_{p\in N_{i}}a_{ip}g_{p}\left(x_{p}\right)\mu_{p}\right), \end{array}$$

where $V_{i}\left(0\right)=0$ and $\nabla C_{i}\left(e_{i}\right)=\partial C_{i}\left(e_{i}\right)/\partial e_{i}$. For the *i*th follower, the local Hamiltonian is

$$\begin{array}{cc} H_{i}\left(e_{i},\mu_{i},\mu_{-i},C_{i}\left(e_{i}\right)\right)=\; & e_{i}^{T}Q_{i}e_{i}+\sum_{p\in \{N_{i},i\}}\mu_{p}^{T}R_{ip}\mu_{p}\\ \; & +\nabla C_{i}^{T}\left(e_{i}\right)\left(F_{i}+c_{i}g_{i}\left(x_{i}\right)\mu_{i}+\sum_{p\in N_{i}}a_{ip}g_{p}\left(x_{p}\right)\mu_{p}\right). \end{array}$$

Define the optimal value function as

(8) $$C_{i}^{*}\left(e_{i}\right)=\min_{\mu_{i}\in A\left(\Omega_{i}\right)}C_{i}\left(e_{i}\right).$$

According to [13], the optimal value function $C_{i}^{*}\left(e_{i}\right)$ satisfies the HJBE as follows

(9) $$0=\min_{\mu_{i}\in A\left(\Omega_{i}\right)}H_{i}\left(e_{i},\mu_{i},\mu_{-i},C_{i}^{*}\left(e_{i}\right)\right).$$

The local OCC protocol is

(10) $$\begin{array}{cc} \mu_{i}^{*}\left(e_{i}\right)=\; & \arg\min_{\mu_{i}\in A\left(\Omega_{i}\right)}H_{i}\left(e_{i},\mu_{i},\mu_{-i},C_{i}^{*}\left(e_{i}\right)\right)\\ =\; & -\frac{1}{2}c_{i}R_{ii}^{-1}g_{i}^{T}\left(x_{i}\right)\nabla C_{i}^{*}\left(e_{i}\right). \end{array}$$

It should be mentioned that the analytical solution of the HJBE is intractable to obtain since $C_{i}^{*}\left(e_{i}\right)$ is unknown. According to [15], the solution of the HJBE is successively approximated through a sequence of iterations with policy evaluation

(11) $$\begin{array}{cc} 0=\; & e_{i}^{T}Q_{i}e_{i}+\sum_{p\in \{N_{i},i\}}\mu_{p}^{(k-1)T}R_{ip}\mu_{p}^{\left(k-1\right)}\\ \; & +\nabla C_{i}^{(k)T}\left(e_{i}\right)\left(F_{i}+c_{i}g_{i}\left(x_{i}\right)\mu_{i}^{\left(k-1\right)}+\sum_{p\in N_{i}}a_{ip}g_{p}\left(x_{p}\right)\mu_{p}^{\left(k-1\right)}\right), \end{array}$$

and policy improvement

(12) $$\begin{array}{c} \mu_{i}^{\left(k\right)}=-\frac{1}{2}c_{i}R_{ii}^{-1}g_{i}^{T}\left(x_{i}\right)\nabla C_{i}^{\left(k\right)}\left(e_{i}\right), \end{array}$$

where (k) represents the *k*th iteration index with $k\in N^{+}$.

From (11), we can see that the policy evaluation requires the accurate mathematical model of (1). However, the accurate mathematical model is always difficult to obtain in practice. To break this bottleneck, the IRL method is developed to relax the requirement of the accurate model in the policy evaluation.

### 3.2. Integral Reinforcement Learning

For $t_{\tau }>0$, (6) can be rewritten as

(13) $$\begin{array}{c} C_{i}\left(e_{i}\left(t\right)\right)=\;\int_{t}^{t+t_{\tau }}\left(e_{i}^{T}\left(\xi \right)Q_{i}e_{i}\left(\xi \right)+\sum_{p\in \{N_{i},i\}}\mu_{p}^{T}\left(\xi \right)R_{ip}\mu_{p}\left(\xi \right)\right)d\xi +C_{i}\left(e_{i}\left(t+t_{\tau }\right)\right). \end{array}$$

Based on the integral Bellman Equation (13), $V_{i}^{*}\left(e_{i}\right)$ and $\mu_{i}^{*}$ satisfy

(14) $$\begin{array}{cc} 0= & \;\int_{t}^{t+t_{\tau }}\left(e_{i}^{T}\left(\xi \right)Q_{i}e_{i}\left(\xi \right)+\sum_{p\in \{N_{i},i\}}\mu_{p}^{*T}\left(\xi \right)R_{ip}\mu_{p}^{*}\left(\xi \right)\right)d\xi \\ \; & +C_{i}^{*}\left(e_{i}\left(t+t_{\tau }\right)\right)-C_{i}^{*}\left(e_{i}\left(t\right)\right). \end{array}$$

Compared to (7), the policy evaluation (14) is not required for the accurate system dynamics in (1).

**Theorem** **1.** *Let $C_{i}^{\left(k\right)}\left(e_{i}\right)\geq 0$, $C_{i}^{\left(k\right)}\left(0\right)=0$ and $\mu_{i}^{\left(k\right)}\in A\left(\Omega_{i}\right)$. $C_{i}^{\left(k\right)}\left(e_{i}\right)$ is the solution of the integral Bellman equation*

(15) $$\begin{array}{cc} 0= & \int_{t}^{t+t_{\tau }}e_{i}^{T}\left(\xi \right)Q_{i}e_{i}\left(\xi \right)d\xi +\int_{t}^{t+t_{\tau }}\sum_{p\in \{N_{i},i\}}\mu_{p}^{(k-1)T}\left(\xi \right)R_{ip}\mu_{p}^{\left(k-1\right)}\left(\xi \right)d\xi \\ \; & +C_{i}^{\left(k\right)}\left(e_{i}\left(t+t_{\tau }\right)\right)-C_{i}^{\left(k\right)}\left(e_{i}\left(t\right)\right), \end{array}$$

*if and only if $C_{i}^{\left(k\right)}\left(e_{i}\right)$ is the only solution of* (11).

**Proof** **of** **Theorem** ** 1.** Considering (11), the time derivative of $C_{i}^{\left(k\right)}\left(e_{i}\right)$ corresponding to (4) is transformed as

(16) $$\begin{array}{cc} \frac{dC_{i}^{\left(k\right)}\left(e_{i}\right)}{dt}=\; & \nabla C_{i}^{\left(k\right)}\left(e_{i}\right)\left(F_{i}+c_{i}g_{i}\left(x_{i}\right)\mu_{i}^{\left(k-1\right)}+\sum_{p\in N_{i}}a_{ip}g_{p}\left(x_{p}\right)\mu_{p}^{\left(k-1\right)}\right)\\ =\; & -e_{i}^{T}Q_{i}e_{i}-\sum_{p\in \{N_{i},i\}}\mu_{p}^{(k-1)T}R_{ip}\mu_{p}^{\left(k-1\right)}. \end{array}$$

Integrate on both sides of (16) within $\left[t,t+t_{\tau }\right]$, that is

(17) $$\begin{array}{cc} C_{i}^{\left(k\right)}\left(e_{i}\left(t+t_{\tau }\right)\right)-C_{i}^{\left(k\right)}\left(e_{i}\left(t\right)\right)= & -\int_{t}^{t+t_{\tau }}e_{i}^{T}\left(\xi \right)Q_{i}e_{i}\left(\xi \right)d\xi \\ \; & -\int_{t}^{t+t_{\tau }}\sum_{p\in \{N_{i},i\}}\mu_{p}^{(k-1)T}\left(\xi \right)R_{ip}\mu_{p}^{\left(k-1\right)}\left(\xi \right)d\xi . \end{array}$$

According to the derivation of (16) and (17), if $C_{i}^{\left(k\right)}\left(e_{i}\right)$ is the solution of (11), $C_{i}^{\left(k\right)}\left(e_{i}\right)$ satisfies the integral Bellman Equation (15). Next, we verify the uniqueness of the solution $C_{i}^{\left(k\right)}\left(e_{i}\right)$.

Supposing that $\Upsilon_{i}^{\left(k\right)}\left(e_{i}\right)$ is another solution of (11) with $\Upsilon_{i}^{\left(k\right)}\left(0\right)=0$. Similar to the mathematical operation of (16), we have

(18) $$\begin{array}{c} \frac{d\Upsilon_{i}^{\left(k\right)}\left(e_{i}\right)}{dt}=-e_{i}^{T}Q_{i}e_{i}-\sum_{p\in \{N_{i},i\}}\mu_{p}^{(k-1)T}R_{ip}\mu_{p}^{\left(k-1\right)}. \end{array}$$

Subtracting (16) into (18) yields

(19) $$\frac{d}{dt}\left(\Upsilon_{i}^{\left(k\right)}\left(e_{i}\right)-C_{i}^{\left(k\right)}\left(e_{i}\right)\right)=0.$$

Solving (19), we have $\Upsilon_{i}^{\left(k\right)}\left(e_{i}\right)-C_{i}^{\left(k\right)}\left(e_{i}\right)=\varsigma_{i}$ with $\varsigma_{i}\in R$ a real constant. For $e_{i}=0$, we have $\varsigma_{i}=\Upsilon_{i}^{\left(k\right)}\left(0\right)-C_{i}^{\left(k\right)}\left(0\right)=0$. That is to say, $\Upsilon_{i}^{\left(k\right)}\left(e_{i}\right)=C_{i}^{\left(k\right)}\left(e_{i}\right)$. One can derive that $C_{i}^{\left(k\right)}\left(e_{i}\right)$ is the unique solution. In summary, $C_{i}^{\left(k\right)}\left(e_{i}\right)$ is the unique solution of (15) if and only if $C_{i}^{\left(k\right)}\left(e_{i}\right)$ is the only solution of (11). □

Theorem 1 reveals that the IRL algorithm with (15) and (12) theoretically equals to the model-based PI algorithm, whose relevant convergence analysis was provided in [15]. Hence, the IRL algorithm can be guaranteed to be convergent.

**Theorem** **2.** Considering the nonlinear MAS with partially unknown dynamic as *(1)*, the local neighborhood containment error dynamic as *(4)* and the optimal value function $C_{i}^{*}\left(e_{i}\right)$ as *(8)*, the closed-loop containment error system is guaranteed to be asymptotically stable under the local OCC protocol *(10)*. Furthermore, the containment control is achieved with a set of the OCC protocols $\{\mu_{1}^{*},\mu_{2}^{*},\ldots ,$$\mu_{N}^{*}\}$ if there is a spanning tree in the directed graph.

**Proof** **of** **Theorem** **2.** Selecting the Lyapunov function candidate as $C_{i}^{*}\left(e_{i}\right)$. Combining (7), (8) and (10), then

(20) $$\begin{array}{cc} \nabla C_{i}^{*T}\left(e_{i}\right)F_{i}= & -\nabla C_{i}^{*T}\left(e_{i}\right)\left(c_{i}g_{i}\left(x_{i}\right)\mu_{i}^{*}+\sum_{p\in N_{i}}a_{ip}g_{p}\left(x_{p}\right)\mu_{p}^{*}\right)\\ \; & -e_{i}^{T}Q_{i}e_{i}-\sum_{p\in \{N_{i},i\}}\mu_{p}^{*T}R_{ip}\mu_{p}^{*}. \end{array}$$

Substituting (20) into the time derivative of $V_{i}^{*}\left(e_{i}\right)$, then

$$\begin{array}{cc} {\dot{C}}_{i}^{*}\left(e_{i}\right)=\; & \nabla C_{i}^{*T}\left(e_{i}\right)\left(F_{i}+c_{i}g_{i}\left(x_{i}\right)\mu_{i}^{*}+\sum_{p\in N_{i}}a_{ip}g_{p}\left(x_{p}\right)\mu_{p}^{*}\right)\\ =\; & -e_{i}^{T}Q_{i}e_{i}-\sum_{p\in \{N_{i},i\}}\mu_{p}^{*T}R_{ip}\mu_{p}^{*}. \end{array}$$

Therefore, ${\dot{C}}_{i}^{*}\left(e_{i}\right)\leq 0$. One can conclude that the closed-loop containment error system (4) is asymptotically stable with the local OCC protocol (10). Since a spanning tree exists in the directed graph, the containment control of the nonlinear MAS with partially unknown dynamic can be achieved. □

### 3.3. Critic NN Implementation

Based on the Stone–Weierstrass approximation theorem, on the compact set $\Omega_{i}$, the optimal function $C_{i}^{*}\left(e_{i}\right)$ and its partial gradient can be established by a critic NN as

(21) $$\begin{array}{cc} C_{i}^{*}\left(e_{i}\right)=\; & \phi_{i}^{*T}\sigma_{i}\left(e_{i}\right)+\omega_{i}\left(e_{i}\right), \end{array}$$

(22) $$\begin{array}{cc} \nabla C_{i}^{*}\left(e_{i}\right)=\; & \nabla \sigma_{i}^{T}\left(e_{i}\right)\phi_{i}^{*}+\nabla \omega_{i}\left(e_{i}\right), \end{array}$$

where $\phi_{i}^{*}\in R^{l_{i}}$ represents the ideal weight, $\sigma_{i}\left(\cdot \right)\in R^{l_{i}}$ represents the activation function, $l_{i}$ represents the number of hidden neurons and $\omega_{i}\left(e_{i}\right)$ stands for the reconstruction error.

Since the ideal weight vector is unknown, the approximation of $C_{i}^{*}\left(e_{i}\right)$ and $\nabla C_{i}^{*}\left(e_{i}\right)$ are expressed as

(23) $$\begin{array}{cc} {\hat{C}}_{i}\left(e_{i}\right) & ={\hat{\phi }}_{i}^{T}\sigma_{i}\left(e_{i}\right),\\ \nabla {\hat{C}}_{i}\left(e_{i}\right) & =\nabla \sigma_{i}^{T}\left(e_{i}\right){\hat{\phi }}_{i}, \end{array}$$

where $\nabla \sigma_{i}\left(e_{i}\right)=\partial \sigma_{i}\left(e_{i}\right)/\partial e_{i}$ and ${\hat{\phi }}_{i}\in R^{l_{i}}$ represents the estimation of $\phi_{i}^{*}$. Then, the local OCC protocol (10) can be approximated by

(24) $${\hat{\mu }}_{i}\left(e_{i}\right)=-\frac{1}{2}c_{i}R_{ii}^{-1}g_{i}^{T}\left(x_{i}\right)\nabla \sigma_{i}^{T}\left(e_{i}\right){\hat{\phi }}_{i}.$$

The approximate local Hamiltonian is

(25) $$\begin{array}{cc} e_{ci}=\; & \int_{t}^{t+t_{\tau }}\left(e_{i}^{T}\left(\xi \right)Q_{i}e_{i}\left(\xi \right)+\sum_{p\in \{N_{i},i\}}{\hat{\mu }}_{p}^{T}\left(\xi \right)R_{ij}{\hat{\mu }}_{p}\left(\xi \right)\right)d\xi \\ \; & +{\hat{\phi }}_{i}^{T}\underset{\theta_{i}}{\underset{︸}{\left(\sigma_{i}\left(e_{i}\left(t+t_{\tau }\right)\right)-\sigma_{i}\left(e_{i}\left(t\right)\right)\right)}}. \end{array}$$

Combining (14) and (21) with (25) yields

(26) $$\begin{array}{cc} e_{ci}= & \int_{t}^{t+t_{\tau }}\left(e_{i}^{T}\left(\xi \right)Q_{i}e_{i}\left(\xi \right)+\sum_{p\in \{N_{i},i\}}{\hat{\mu }}_{p}^{T}\left(\xi \right)R_{ip}{\hat{\mu }}_{p}\left(\xi \right)\right)d\xi \\ & -\int_{t}^{t+t_{\tau }}\left(e_{i}^{T}\left(\xi \right)Q_{i}e_{i}\left(\xi \right)+\sum_{p\in \{N_{i},i\}}\mu_{p}^{*T}\left(\xi \right)R_{ip}\mu_{p}^{*}\left(\xi \right)\right)d\xi \\ \; & +{\hat{\phi }}_{i}^{T}\theta_{i}-\phi_{i}^{*T}\theta_{i}-\omega_{i}\left(e_{i}\left(t+t_{\tau }\right)\right)+\omega_{i}\left(e_{i}\left(t\right)\right)\\ = & \int_{t}^{t+t_{\tau }}\sum_{p\in \{N_{i},i\}}{\left({\hat{\mu }}_{p}\left(\xi \right)+\mu_{p}^{*}\left(\xi \right)\right)}^{T}R_{ip}\left({\hat{\mu }}_{p}\left(\xi \right)-\mu_{p}^{*}\left(\xi \right)\right)d\xi \\ \; & -{\tilde{\phi }}_{i}\theta_{i}-\omega_{i}\left(e_{i}\left(t+t_{\tau }\right)\right)+\omega_{i}\left(e_{i}\left(t\right)\right)\\ = & -{\tilde{\phi }}_{i}\theta_{i}+\Phi_{i}, \end{array}$$

where ${\tilde{\phi }}_{i}=\phi_{i}^{*}-{\hat{\phi }}_{i}$ represents the weight estimation error and $\Phi_{i}=\int_{t}^{t+t_{\tau }}\sum_{p\in \{N_{i},i\}}{\left({\hat{\mu }}_{p}\left(\xi \right)+\mu_{p}^{*}\left(\xi \right)\right)}^{T}R_{ip}\left({\hat{\mu }}_{p}\left(\xi \right)-\mu_{p}^{*}\left(\xi \right)\right)d\xi -\omega_{i}\left(e_{i}\left(t+t_{\tau }\right)\right)+\omega_{i}\left(e_{i}\left(t\right)\right)$.

**Assumption** **2.** *$\Phi_{i}$ is bounded by $\eta_{i}$, i.e., $∥\Phi_{i}∥\leq \eta_{i}$ with $\eta_{i}>0$.*

In order to tune ${\hat{\phi }}_{i}$, the steepest descent algorithm is employed to minimize $E_{ci}=\frac{1}{2}e_{ci}^{2}$. A modified updating law of ${\hat{\phi }}_{i}$ is

(27) $${\dot{\hat{\phi }}}_{i}=-l_{ci}\frac{\theta_{i}}{{\left(1+\theta_{i}^{T}\theta_{i}\right)}^{2}}\left(e_{ci}-{\hat{\eta }}_{i}\right)$$

where $l_{ci}>0$ and ${\hat{\eta }}_{i}$, the estimation of $\eta_{i}$, can be updated by

(28) $${\dot{\hat{\eta }}}_{i}=l_{si}\frac{{\tilde{\phi }}_{i}^{T}\theta_{i}}{{\left(1+\theta_{i}^{T}\theta_{i}\right)}^{2}},$$

where $l_{si}>0$ is a design constant. Considering (26) and (27), the weight estimation error is updated by

(29) $${\dot{\tilde{\phi }}}_{i}=-l_{ci}\frac{\theta_{i}}{{\left(1+\theta_{i}^{T}\theta_{i}\right)}^{2}}\left({\tilde{\phi }}^{T}\theta_{i}-\Phi_{i}+{\hat{\eta }}_{i}\right).$$

**Theorem** **3.** *Considering the nonlinear MAS with partially unknown dynamic as *(1)*, the local neighborhood containment error dynamic as *(4)* and the critic NN with the modified updating laws *(27)* and *(28)*, then ${\tilde{\phi }}_{i}$ is guaranteed to be asymptotically stable.*

**Proof** **of** **Theorem** **3.** Define ${\tilde{\eta }}_{i}=\eta_{i}-{\hat{\eta }}_{i}$. Choose the Lyapunov function candidate as

(30) $$\Xi_{ci}=\frac{1}{2l_{ci}}{\tilde{\phi }}_{i}^{T}{\tilde{\phi }}_{i}+\frac{1}{2l_{si}}{\tilde{\eta }}_{i}^{2}.$$

According to (28), ${\tilde{\eta }}_{i}$ is updated by

(31) $${\dot{\tilde{\eta }}}_{i}=-l_{si}\frac{{\tilde{\phi }}_{i}^{T}\theta_{i}}{{\left(1+\theta_{i}^{T}\theta_{i}\right)}^{2}}.$$

Considering (29) and (31), the time derivative of (30) is

(32) $$\begin{array}{cc} {\dot{\Xi }}_{ci}=\; & \frac{1}{l_{ci}}{\tilde{\phi }}_{i}^{T}{\dot{\tilde{\phi }}}_{i}+\frac{1}{l_{si}}{\tilde{\eta }}_{i}{\dot{\tilde{\eta }}}_{i}\\ =\; & -\frac{{\tilde{\phi }}_{i}^{T}\theta_{i}}{{\left(1+\theta_{i}^{T}\theta_{i}\right)}^{2}}\left({\tilde{\phi }}^{T}\theta_{i}-\Phi_{i}+{\hat{\eta }}_{i}\right)-\frac{{\tilde{\phi }}_{i}^{T}\theta_{i}}{{\left(1+\theta_{i}^{T}\theta_{i}\right)}^{2}}{\tilde{\eta }}_{i}\\ =\; & -{\tilde{\phi }}_{i}^{T}\Psi_{i}{\tilde{\phi }}_{i}+\frac{{\tilde{\phi }}_{i}^{T}\theta_{i}}{{\left(1+\theta_{i}^{T}\theta_{i}\right)}^{2}}\left(\Phi_{i}-{\hat{\eta }}_{i}-{\tilde{\eta }}_{i}\right), \end{array}$$

where $\Psi_{i}=\theta_{i}\theta_{i}^{T}/{\left(1+\theta_{i}^{T}\theta_{i}\right)}^{2}$. According to Assumption 2, (32) is derived as

$$\begin{array}{cc} {\dot{\Xi }}_{ci}\leq \; & -\lambda_{\min}\left(\Psi_{i}\right){∥{\tilde{\phi }}_{i}∥}^{2}+\frac{{\tilde{\phi }}_{i}^{T}\theta_{i}}{{\left(1+\theta_{i}^{T}\theta_{i}\right)}^{2}}\left(∥\Phi_{i}∥-\eta_{i}\right)\\ \leq \; & -\lambda_{\min}\left(\Psi_{i}\right){∥{\tilde{\phi }}_{i}∥}^{2}. \end{array}$$

It indicates ${\dot{\Xi }}_{ci}\leq 0$. Therefore, one can conclude that ${\tilde{\phi }}_{i}$ is ensured to be asymptotically stable. □

**Figure 1:**
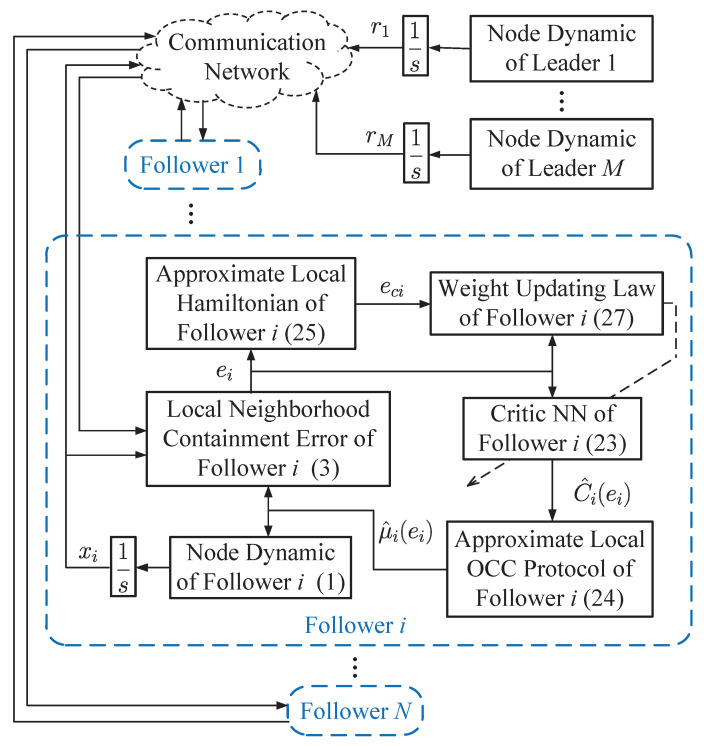
Structural diagram of the developed IRL-based OCC scheme.

Under the framework of the critic-only architecture, the IRL-based OCC scheme is presented. For each follower, the local neighborhood containment error (3) is established by communicating with its neighbors and the leaders. The value function of each follower is approximated by the critic NN (23), whose weights are tuned by a modified weight updating law (27). Based on (1), (3) and (23), the local OCC protocol (24) is obtained. The structural diagram of the developed IRL-based OCC scheme is shown in Figure 1.

**Remark 1.**  *In the actor–critic architecture, the optimal value function and the optimal control policy are approximated by a critic NN and an actor NN, respectively. While for the critic-only architecture, the optimal value function is approximated by a critic NN and the optimal control policy is directly obtained by combining (10) and (22). Hence, the critic-only architecture keeps the same performance as the actor–critic one. In contrast, the critic-only architecture utilizes a single critic NN only, which implies that the control structure is simplified and the computation burden is reduced.*

### 3.4. Stability Analysis

**Assumption** **3.** $\phi_{i}^{*}$, ${\tilde{\phi }}_{i}$, $\nabla \sigma_{i}\left(\cdot \right)$ and $\nabla \omega_{i}\left(\cdot \right)$ are norm-bounded, i.e.,

$$\begin{array}{c} ∥\phi_{i}^{*}∥\leq \phi_{iM},\;\;\;∥{\tilde{\phi }}_{i}∥\leq {\bar{\phi }}_{iM},\;\;\;∥\nabla \sigma_{i}\left(\cdot \right)∥\leq {\bar{\sigma }}_{iM},\;\;\;∥\nabla \omega_{i}\left(\cdot \right)∥\leq {\bar{\omega }}_{iM},\;\;\;∥g_{i}\left(\cdot \right)∥\leq {\bar{g}}_{iM}, \end{array}$$

*where $\phi_{iM}$, ${\bar{\phi }}_{iM}$, ${\bar{\sigma }}_{iM}$, ${\bar{\omega }}_{iM}$ and ${\bar{g}}_{iM}$ are positive constants.*

**Theorem 4.**  *Considering the nonlinear MAS with partially unknown dynamics as (1), the local neighborhood containment error dynamic as (4), the optimal value function as (8) and the critic NN which is updated by (27) and (28), the local containment control protocol (24) can guarantee the closed-loop containment error system (4) to be UUB.*

**Proof** **of** **Theorem** **4.** The Lyapunov function candidate is chosen as

(33) $$\Xi_{i}=C_{i}^{*}\left(e_{i}\right).$$

Considering (20), (21) and Assumption 3, the time derivative of (33) corresponding to (4) is

(34) $$\begin{array}{cc} {\dot{\Xi }}_{i}=\; & {\dot{C}}_{i}^{*}\left(e_{i}\right)\\ =\; & \nabla C_{i}^{*T}\left(e_{i}\right)\left(F_{i}+c_{i}g_{i}\left(x_{i}\right){\hat{\mu }}_{i}+\sum_{p\in N_{i}}a_{ip}g_{p}\left(x_{p}\right){\hat{\mu }}_{p}\right)\\ =\; & \nabla C_{i}^{*T}\left(e_{i}\right)\left(c_{i}g_{i}\left(x_{i}\right)\left({\hat{\mu }}_{i}-\mu_{i}^{*}\right)+\sum_{p\in N_{i}}a_{ip}g_{p}\left(x_{p}\right)\left({\hat{\mu }}_{p}-\mu_{p}^{*}\right)\right)-e_{i}^{T}Q_{i}e_{i}-\sum_{p\in \{N_{i},i\}}\mu_{p}^{*T}R_{ip}\mu_{p}^{*}\\ \leq \; & ∥\nabla C_{i}^{*T}\left(e_{i}\right)∥\left(∥c_{i}g_{i}\left(x_{i}\right)\left({\hat{\mu }}_{i}-\mu_{i}^{*}\right)∥+\sum_{p\in N_{i}}∥a_{ip}g_{p}\left(x_{p}\right)\left({\hat{\mu }}_{p}-\mu_{p}^{*}\right)∥\right)-\lambda_{\min}\left(Q_{i}\right){∥e_{i}∥}^{2}\\ \leq \; & \left({\bar{\sigma }}_{iM}\phi_{iM}+{\bar{\omega }}_{iM}\right)\left(c_{i}{\bar{g}}_{iM}∥{\hat{\mu }}_{i}-\mu_{i}^{*}∥+\sum_{p\in N_{i}}a_{ip}{\bar{g}}_{pM}∥{\hat{\mu }}_{p}-\mu_{p}^{*}∥\right)-\lambda_{\min}\left(Q_{i}\right){∥e_{i}∥}^{2}. \end{array}$$

Notice that

$$\begin{array}{cc} ∥{\hat{\mu }}_{i}-\mu_{i}^{*}∥=\; & ∥-\frac{1}{2}R_{ii}^{-1}c_{i}g_{i}^{T}\left(x_{i}\right)\nabla \sigma_{i}^{T}\left(e_{i}\right){\hat{\phi }}_{i}+\frac{1}{2}R_{ii}^{-1}c_{i}g_{i}^{T}\left(x_{i}\right)\left(\nabla \sigma_{i}^{T}\left(e_{i}\right)\phi_{i}^{*}+\nabla \omega_{i}\left(e_{i}\right)\right)∥\\ =\; & ∥\frac{1}{2}R_{ii}^{-1}c_{i}g_{i}^{T}\left(x_{i}\right)\left(\nabla \sigma_{i}^{T}\left(e_{i}\right){\tilde{\phi }}_{i}+\nabla \omega_{i}\left(e_{i}\right)\right)∥\\ \leq \; & \frac{c_{i}{\bar{g}}_{iM}}{2∥R_{ii}∥}\left({\bar{\sigma }}_{iM}{\bar{\phi }}_{iM}+{\bar{\omega }}_{iM}\right). \end{array}$$

Then, (34) becomes

(35) $$\begin{array}{cc} {\dot{\Xi }}_{i}\leq & \left({\bar{\sigma }}_{iM}\phi_{iM}+{\bar{\omega }}_{iM}\right)\left(\frac{c_{i}^{2}{\bar{g}}_{iM}^{2}}{2∥R_{ii}∥}\left({\bar{\sigma }}_{iM}{\bar{\phi }}_{iM}+{\bar{\omega }}_{iM}\right)+\sum_{p\in N_{i}}\frac{c_{p}a_{ip}{\bar{g}}_{pM}^{2}}{2∥R_{pp}∥}\left({\bar{\sigma }}_{pM}{\bar{\phi }}_{pM}+{\bar{\omega }}_{pM}\right)\right)\\ \; & -\lambda_{\min}\left(Q_{i}\right){∥e_{i}∥}^{2}. \end{array}$$

Let $\Pi_{i1}=\frac{c_{i}^{2}{\bar{g}}_{iM}^{2}}{2∥R_{ii}∥}\left({\bar{\sigma }}_{iM}{\bar{\phi }}_{iM}+{\bar{\omega }}_{iM}\right)+\sum_{p\in N_{i}}\frac{c_{p}a_{ip}{\bar{g}}_{pM}^{2}}{2∥R_{pp}∥}$$\left({\bar{\sigma }}_{pM}{\bar{\phi }}_{pM}+{\bar{\omega }}_{pM}\right)$. Thus, (35) turns to

$$\begin{array}{cc} {\dot{\Xi }}_{i}\leq \; & \underset{\Pi_{i2}}{\underset{︸}{\left({\bar{\sigma }}_{iM}\phi_{iM}+{\bar{\omega }}_{iM}\right)\Pi_{i1}}}-\lambda_{\min}\left(Q_{i}\right){∥e_{i}∥}^{2}\\ =\; & \Pi_{i2}-\lambda_{\min}\left(Q_{i}\right){∥e_{i}∥}^{2}. \end{array}$$

It shows ${\dot{L}}_{i2}<0$ if $e_{i}$ lies outside the compact set

$$\Omega_{e_{i}}=\left\{e_{i}:∥e_{i}∥\leq \sqrt{\frac{\Pi_{i2}}{\lambda_{\min}\left(Q_{i}\right)}}\right\}.$$

Therefore, the closed-loop containment error system (4) is UUB under the local containment control protocol (24). □

**Remark** **2.** *In Assumption 1, we know that the nonlinear functions f(x) and $g_{i}\left(x\right)$ are Lipschitz continuous on a compact set $\Omega_{i}$ containing the origin, f(0)=0. It indicates that the developed control scheme is effective in a compact set $\Omega_{i}$. If the system states are outside this compact set, this scheme might be invalid. In Theorem 4, we analyzed the system stability within such a compact set via the Lyapunov direct method, which means the closed-loop system is stable in the compact set under the developed IRL-based OCC scheme.*

## 4. Simulation Study

This section provides two simulation examples to support the developed IRL-based OCC scheme.

### 4.1. Example 1

**Figure 2:**
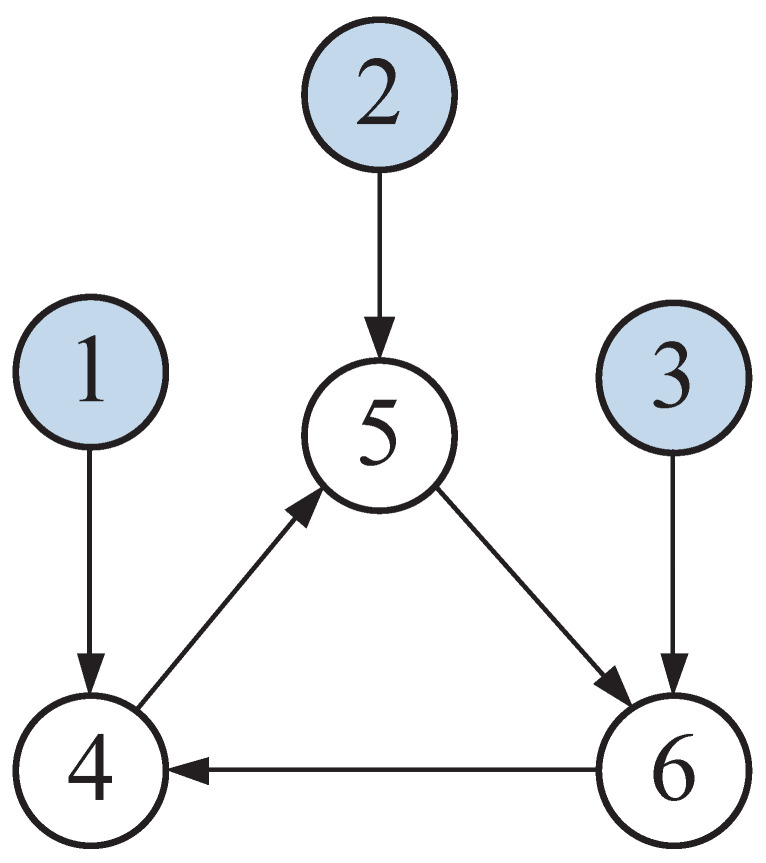
The directed topology of example 1.

Consider a six-node graph network connected by three leader nodes. The directed topology of the graph is displayed in Figure 2.

As displayed in Figure 2, nodes 1–3 stand for the leaders 1–3 and nodes 4–6 represent the followers 1–3. In (3), the edge weights and pinning gains were set to 0.5. The node dynamic of the *j*th leader is described as ${\dot{r}}_{j}=\bar{A}r_{j}$, where $r_{j}={\left[r_{j1},r_{j2}\right]}^{T}\in R^{2}$ represents the state vector, j=1,2,3 and

$$\bar{A}=\left[\begin{array}{cc} 0.1 & -1\\ 1 & -0.1 \end{array}\right].$$

For the *i*th follower, the node dynamic is formulated as ${\dot{x}}_{i}=\bar{A}x_{i}+{\bar{B}}_{i}\mu_{i}$, where $x_{i}={\left[x_{i1},x_{i2}\right]}^{T}\in R^{2}$ and $\mu_{i}\in R$ with i=1,2,3, ${\bar{B}}_{1}={\left[-1.5,1\right]}^{T}$, ${\bar{B}}_{2}={\left[-1,1\right]}^{T}$ and ${\bar{B}}_{3}={\left[-1,-0.5\right]}^{T}$. The local neighborhood containment error vector $e_{i}={\left[e_{i1},e_{i2}\right]}^{T}\in R^{2}$ is calculated by (3).

In the simulation, $C_{i}\left(e_{i}\right)$ was reconstructed by a critic NN with a 2–5–1 structure. The activation function was described as $\sigma_{i}\left(e_{i}\right)={\left[e_{i1}^{2},e_{i1}e_{i2},e_{i2}^{2},e_{i1}^{2}e_{i2},e_{i2}^{2}e_{i1}\right]}^{T}$. The initialization of the node dynamics were characterized as $x_{1}\left(0\right)={\left[0.50,-1.00\right]}^{T}$, $x_{2}\left(0\right)={\left[1.00,-0.50\right]}^{T}$, $x_{3}\left(0\right)={\left[0.80,-0.30\right]}^{T}$, $r_{1}\left(0\right)={\left[0.62,0.83\right]}^{T}$, $r_{2}\left(0\right)={\left[0.45,0.40\right]}^{T}$ and $r_{3}\left(0\right)={\left[0.30,0.22\right]}^{T}$. The related parameters were chosen as $Q_{i}=5I_{2}$, $R_{ip}=R_{ii}=1$, $l_{ci}=0.1$ and $l_{si}=0.1$.

**Figure 3:**
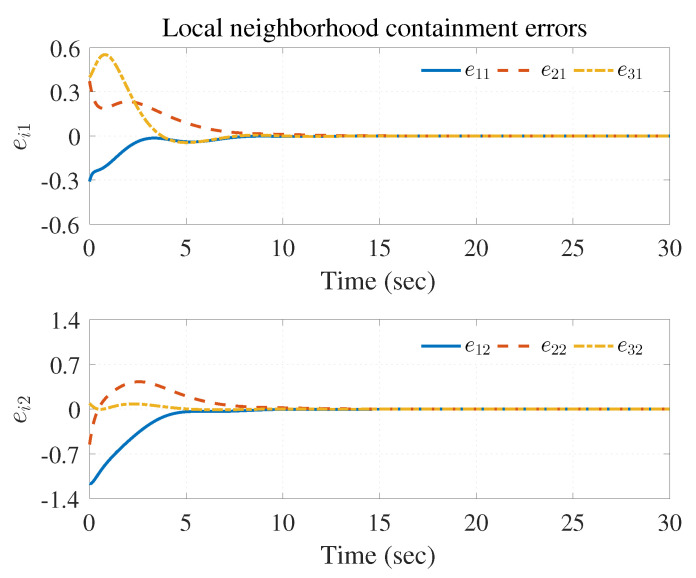
Local neighborhood containment errors $e_{i}$.

**Figure 4:**
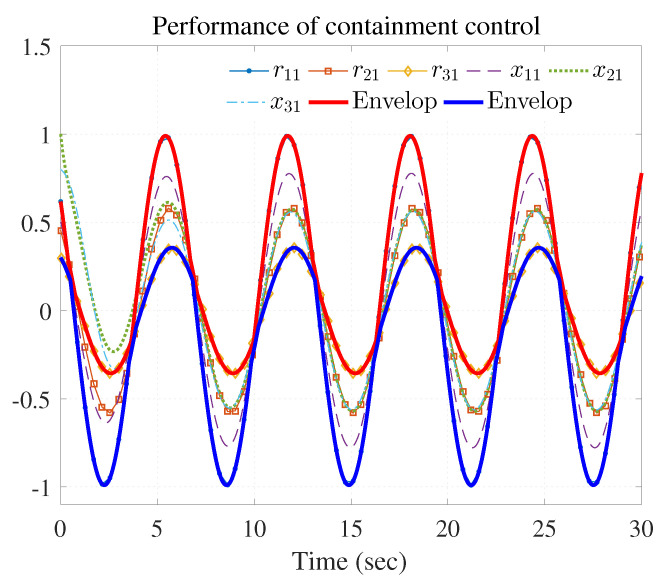
Performance of containment control ($r_{j1}$ and $x_{i1}$).

**Figure 5:**
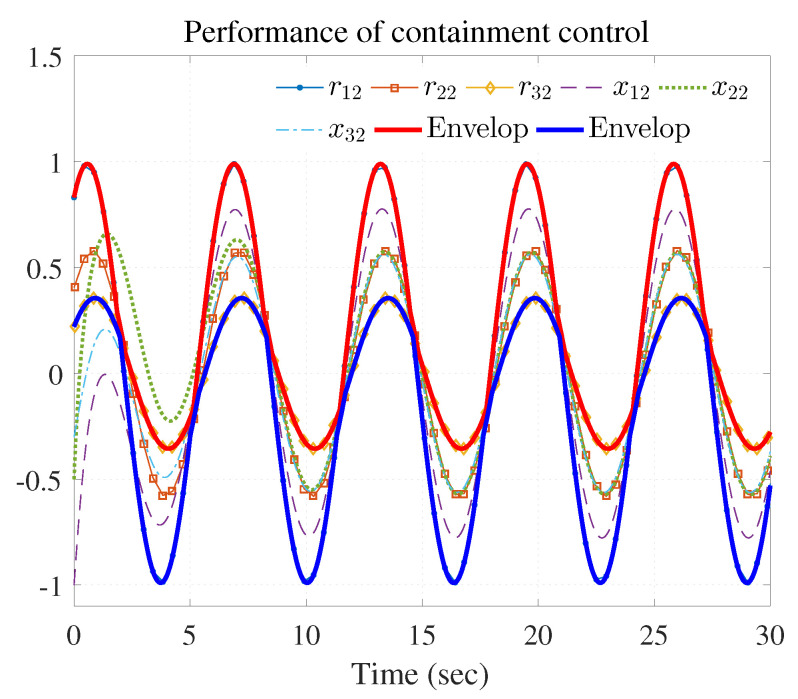
Performance of containment control ($r_{j2}$ and $x_{i2}$).

**Figure 6:**
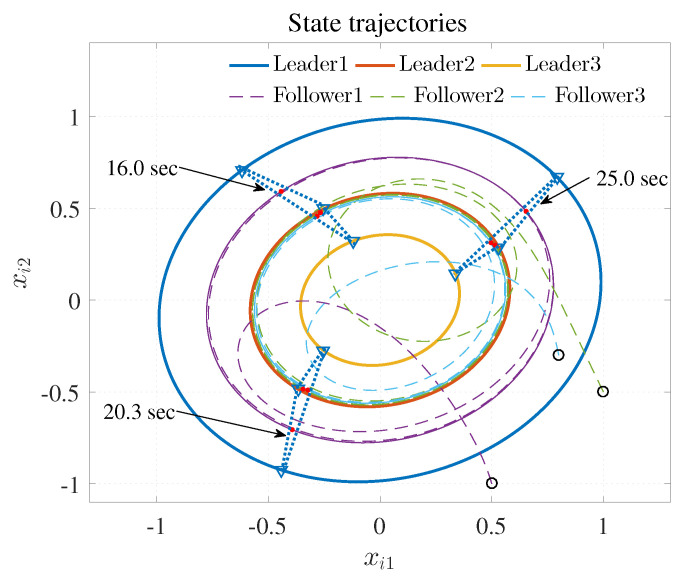
State trajectories.

The simulation results are shown in Figure 3, Figure 4 and Figure 5 using the developed IRL-based OCC protocols. The evolution procedure of the local neighborhood containment errors for triple followers is shown in Figure 3, which indicates that the local neighborhood containment errors were regulated to zero under the developed control protocols. Thus, the containment control of MAS could be reached. Figure 4 and Figure 5 depict the state curves of the leaders and the followers, where all followers moved and stayed within the region formed by the envelope curves. It implies that the satisfactory performance of the containment control was acquired. The state curves of the followers and the leaders are displayed as 2-D phase plane plot in Figure 6 and the region enveloped by the three leaders $\upsilon_{1},\upsilon_{2}$ and $\upsilon_{3}$ is shown at three different instants (t=16.0s,20.3s and 25.0s). We can observe from Figure 6 that the followers converged to the convex hull.

### 4.2. Example 2

Consider the nonlinear MAS consisting of three single-link robot arms and triple leader nodes. A rigid link is attached to each robot arm via a gear train to a direct current motor [28]. In Figure 2, the directed topology among these robot arms is shown. We chose the values of all edge weights and pinning gain as 1.

The state trajectories of the leaders is given by $r_{1}={\left[0.6\sin\left(t\right),0.6\cos\left(t\right)\right]}^{T}$, $r_{2}={\left[0.4\sin\left(t+\frac{\pi }{6}\right),0.4\cos\left(t+\frac{\pi }{6}\right)\right]}^{T}$ and $r_{3}={\left[0.2\sin\left(t-\frac{\pi }{6}\right),0.2\cos\left(t-\frac{\pi }{6}\right)\right]}^{T}$. The single-link robot arm for each follower can be described as

(36) $$J{\ddot{z}}_{i}+\bar{B}{\dot{z}}_{i}+\bar{M}gl\sin\left(z_{i}\right)=u_{i},$$

where $J=9\;\mathrm{kg}\cdot m{}^{2}$, $\bar{B}=30.5$, $\bar{M}=1\;\mathrm{kg}$, l=1m, $g=9.8\;m/s{}^{2}$ and i=1,2,3. The notations of the model (36) are defined in Table 1.

Define $x_{i}={\left[x_{i1},x_{i2}\right]}^{T}={\left[z_{i},{\dot{z}}_{i}\right]}^{T}\in R^{2}$ and $\mu_{i}=u_{i}$. For the *i*th follower, the model (36) can be rewritten as

(37) $$\begin{array}{cc} \left[\begin{array}{c} {\dot{x}}_{i1}\\ {\dot{x}}_{i2} \end{array}\right]= & \left[\begin{array}{c} x_{i2}\\ -\frac{\bar{M}gl}{J}\sin\left(x_{i1}\right)-\frac{\bar{B}}{J}x_{i2} \end{array}\right]+\left[\begin{array}{c} 0\\ \frac{1}{J} \end{array}\right]\mu_{i}. \end{array}$$

Similar to Example Section 4.1, the local neighborhood containment error vector was given as $e_{i}={\left[e_{i1},e_{i2}\right]}^{T}\in R^{2}$.

The critic NN structures and the related activation functions were initialized as in Example Section 4.1. The critic NN weights were initialized as the random values within (0,36) and the parameters of initialization and control were chosen as $r_{1}\left(0\right)={\left[0,0.6\right]}^{T}$, $r_{2}\left(0\right)={\left[0.4\sin\left(\frac{\pi }{6}\right),0.4\cos\left(\frac{\pi }{6}\right)\right]}^{T}$, $r_{3}\left(0\right)={\left[0.2\sin\left(-\frac{\pi }{6}\right),0.2\cos\left(-\frac{\pi }{6}\right)\right]}^{T}$, $x_{1}\left(0\right)={\left[0.8,0.1\right]}^{T}$, $x_{2}\left(0\right)={\left[0.6,0.5\right]}^{T}$, $x_{3}\left(0\right)={\left[0.7,-0.3\right]}^{T}$, $Q_{ip}=18I_{n}$, $R_{ip}=5$, $t_{\tau }=0.1\;s$, $l_{ci}=0.1$ and $l_{si}=0.1$.

**Figure 7:**
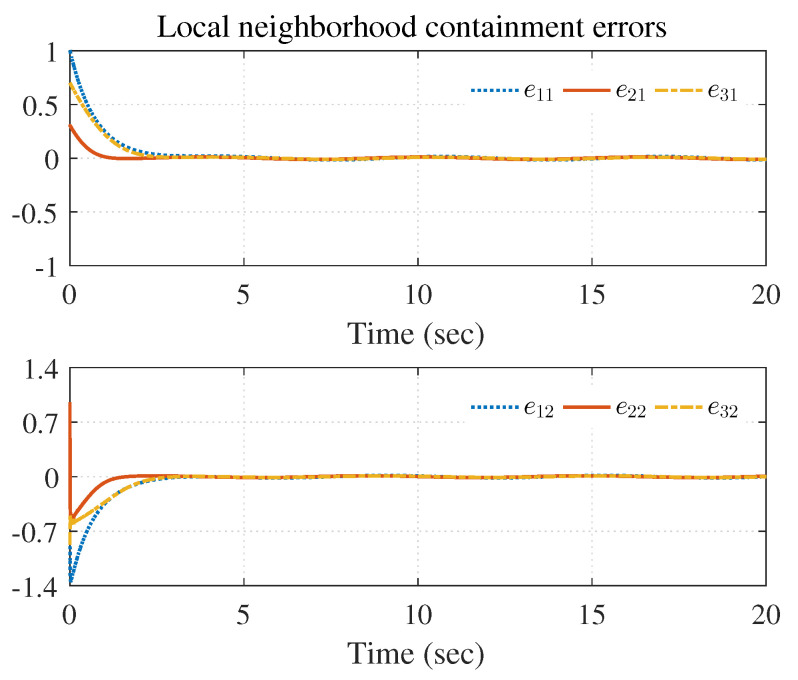
Local neighborhood containment errors of triple followers.

**Figure 8:**
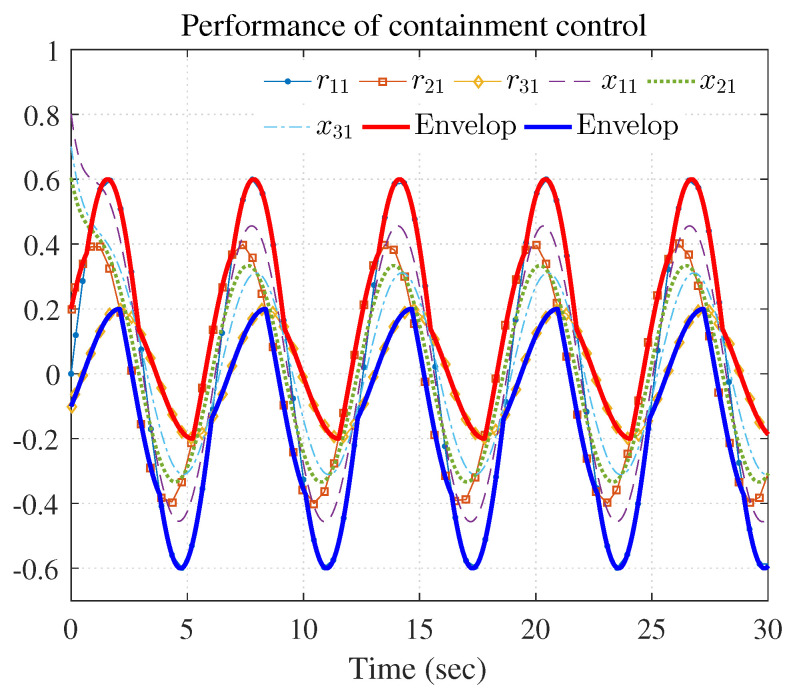
Performance of containment control ($r_{j1}$ and $x_{i1}$).

**Figure 9:**
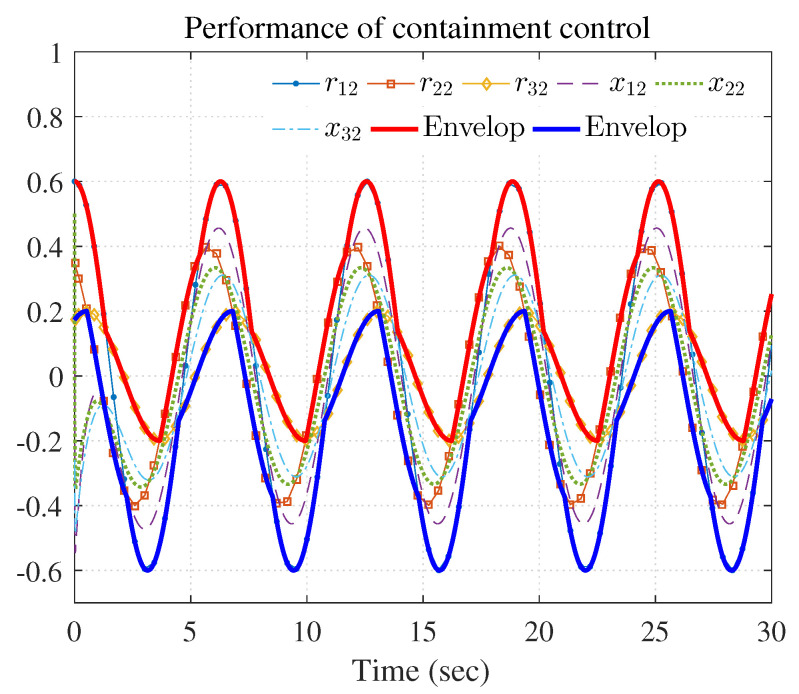
Performance of containment control ($r_{j2}$ and $x_{i2}$).

**Figure 10:**
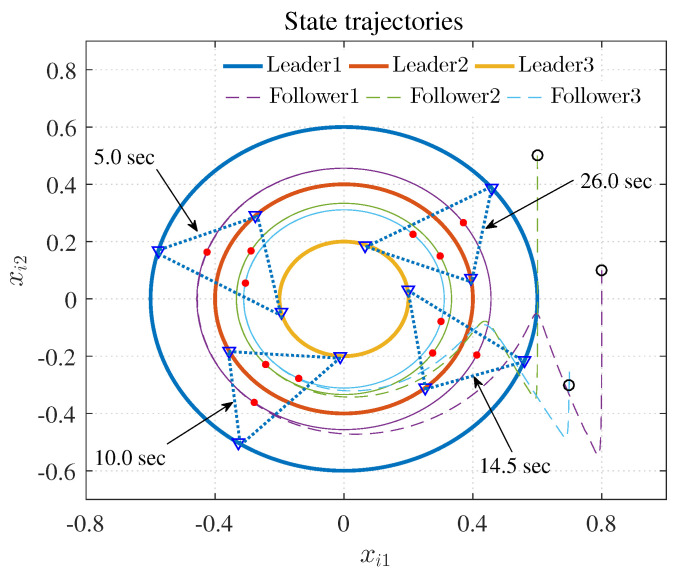
State trajectories.

**Figure 11:**
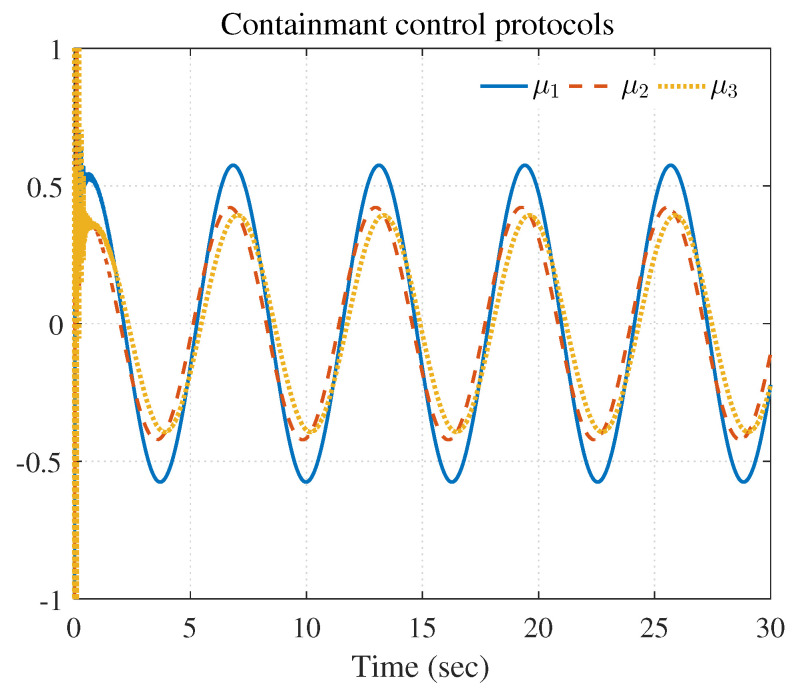
Containment control inputs of triple followers.

Figure 7, Figure 8, Figure 9, Figure 10 and Figure 11 show the simulation results. The local neighborhood containment errors converged to a small region around zero as depicted in Figure 7, which shows that the containment control of the nonlinear MAS was achieved. In Figure 8 and Figure 9, it can be found that the state trajectories of single-link robot arms (36) entered and stayed within the region enveloped by the leader nodes as the time progressed, which indicated the satisfactory performance of the developed scheme. The evolution curves of all agents are illustrated as the 2-D phase plane plot in Figure 10. We can see that the convex hull formed by the leaders $\upsilon_{1},\upsilon_{2}$ and $\upsilon_{3}$ contains the followers at the time instants t=5.0s,10.0s,14.5s and 26.0s, which implies that the followers converged to the convex hull. Figure 11 describes the curves of the containment control inputs, which shows the regulation process of the containment error system.

## 5. Conclusions

This paper investigated the OCC problem of nonlinear MASs with partially unknown dynamics via the IRL method. Based on the IRL method, the integral Bellman equation was constructed to relax the requirement of the drift dynamics. The proposed control algorithm was guaranteed to converge by analyzing the convergence of IRL. With the aid of the universal approximation capability of the NN, the solution of the HJBE was acquired by a critic NN with a modified weight-updating law which guaranteed the asymptotical stability of the weight error dynamics. By using the Lyapunov stability theorem, we showed that the closed-loop containment error system was UUB. From the simulation results of two examples, the effectiveness of the proposed IRL-based OCC scheme was illustrated. In the considered MASs, the information among all agents was transmitted by a desired communication network, which is always confronted with some security issues, such as attacks and packet dropouts. The focus of our future work is to develop a novel distributed resilient containment control for the MASs subjected to attacks and packet dropouts.

## Figures and Tables

**Table 1:** Notations of the single-link robot arm.

Symbol	Notation
$z_{i}$	Link angle
${\dot{z}}_{i}$	Angular velocity of the link
J	Total rotational inertia of the link and motor
$\bar{B}$	Overall damping coefficient
$\bar{M}$	Total mass of the link
*l*	Distant from joint axis to mass center of the link
$u_{i}$	Command generator

## Data Availability

Data is contained within this manuscript.

## References

[1] Jimenez A.F., Cardenas P.F., Jimenez F. (2022). Intelligent IoT-multiagent precision irrigation approach for improving water use efficiency in irrigation systems at farm and district scales. Comp. Electr. Agric..

[2] Vallejo D., Castro-Schez J., Glez-Morcillo C., Albusac J. (2020). Multi-agent architecture for information retrieval and intelligent monitoring by UAVs in known environments affected by catastrophes. Eng. Appl. Artif. Intell..

[3] Liu Y., Wang Y., Li Y., Gooi H.B., Xin H. (2021). Multi-agent based optimal scheduling and trading for multi-microgrids integrated with urban transportation networks. IEEE Trans. Power. Syst..

[4] Deng Q., Peng Y., Qu D., Han T., Zhan X. (2022). Neuro-adaptive containment control of unmanned surface vehicles with disturbance observer and collision-free. ISA Trans..

[5] Hamani N., Jamont J.P., Occello M., Ben-Yelles C.B., Lagreze A., Koudil M. (2018). A multi-cooperative-based approach to manage communication in wireless instrumentation systems. IEEE Syst. J..

[6] Ren W., Beard R.W. (2005). Consensus seeking in multiagent systems under dynamically changing interaction topologies. IEEE Trans. Autom. Control.

[7] Luo K., Guan Z.H., Cai C.X., Zhang D.X., Lai Q., Xiao J.W. (2019). Coordination of nonholonomic mobile robots for diffusive threat defense. J. Frankl. Inst..

[8] Yu Z., Liu Z., Zhang Y., Qu Y., Su C.Y. (2020). Distributed finite-time fault-tolerant containment control for multiple unmanned aerial vehicles. IEEE Trans. Neural Netw. Learn. Syst..

[9] Li Y., Qu F., Tong S. (2021). Observer-based fuzzy adaptive finite-time containment control of nonlinear multiagent systems with input delay. IEEE Trans. Cybern..

[10] Li Z., Xue H., Pan Y., Liang H. (2022). Distributed adaptive event-triggered containment control for multi-agent systems under a funnel function. Int. J. Robust Nonlinear Control.

[11] Li Y., Liu M., Lian J., Guo Y. (2022). Collaborative optimal formation control for heterogeneous multi-agent systems. Entropy.

[12] Zhao L., Yu J., Shi P. (2021). Command filtered backstepping-based attitude containment control for spacecraft formation. IEEE Trans. Syst. Man Cybern. Syst..

[13] Liu D., Wei Q., Wang D., Yang X., Li H. (2017). Adaptive Dynamic Programming with Applications in Optimal Control.

[14] Bellman R.E. (1957). Dynamic Programming.

[15] Abu-Khalaf M., Lewis F.L. (2005). Nearly optimal control laws for nonlinear systems with saturating actuators using a neural network HJB approach. Automatica.

[16] Liu D., Xue S., Zhao B., Luo B., Wei Q. (2021). Adaptive dynamic programming for control: A survey and recent advances. IEEE Trans. Syst. Man. Cybern. Syst..

[17] Vamvoudakis K.G., Lewis F.L., Hudas G.R. (2012). Multi-agent differential graphical games: Online adaptive learning solution for synchronization with optimality. Automatica.

[18] Zhang H., Zhang J., Yang G., Luo Y. (2015). Leader-based optimal coordination control for the consensus problem of multiagent differential games via fuzzy adaptive dynamic programming. IEEE Trans. Fuzzy. Syst..

[19] Zhao W., Zhang H. (2019). Distributed optimal coordination control for nonlinear multi-agent systems using event-triggered adaptive dynamic programming method. ISA Trans..

[20] Cui J., Pan Y., Xue H., Tan L. (2022). Simplified optimized finite-time containment control for a class of multi-agent systems with actuator faults. Nonlinear Dyn..

[21] Xu J., Wang L., Liu Y., Xue H. (2022). Event-triggered optimal containment control for multi-agent systems subject to state constraints via reinforcement learning. Nonlinear Dyn..

[22] Xiao W., Zhou Q., Liu Y., Li H., Lu R. (2022). Distributed reinforcement learning containment control for multiple nonholonomic mobile robots. IEEE Trans. Circuits Syst. I Reg. Papers.

[23] Chen C., Lewis F.L., Xie K., Xie S., Liu Y. (2020). Off-policy learning for adaptive optimal output synchronization of heterogeneous multi-agent systems. Automatica.

[24] Yu D., Ge S.S., Li D., Wang P. (2021). Finite-horizon robust formation-containment control of multi-agent networks with unknown dynamics. Neurocomputing.

[25] Zuo S., Song Y., Lewis F.L., Davoudi A. (2018). Optimal robust output containment of unknown heterogeneous multiagent system using off-policy reinforcement learning. IEEE Trans. Cybern..

[26] Mazouchi M., Naghibi-Sistani M.B., Hosseini Sani S.K., Tatari F., Modares H. (2019). Observer-based adaptive optimal output containment control problem of linear heterogeneous Multiagent systems with relative output measurements. Int. J. Adapt. Control Signal Process..

[27] Yang Y., Modares H., Wunsch D.C., Yin Y. (2019). Optimal containment control of unknown heterogeneous systems with active leaders. IEEE Trans. Control Syst. Technol..

[28] Zhang H., Lewis F.L., Qu Z. (2012). Lyapunov, adaptive, and optimal design techniques for cooperative systems on directed communication graphs. IEEE Trans. Ind. Electron..

